# Assessing the quality of steady-state visual-evoked potentials for moving humans using a mobile electroencephalogram headset

**DOI:** 10.3389/fnhum.2014.00182

**Published:** 2014-03-31

**Authors:** Yuan-Pin Lin, Yijun Wang, Chun-Shu Wei, Tzyy-Ping Jung

**Affiliations:** Swartz Center for Computational Neuroscience, Institute for Neural Computation, University of CaliforniaSan Diego, CA, USA

**Keywords:** EEG, BCI, mobile EEG system, SSVEP, moving humans

## Abstract

Recent advances in mobile electroencephalogram (EEG) systems, featuring non-prep dry electrodes and wireless telemetry, have enabled and promoted the applications of mobile brain-computer interfaces (BCIs) in our daily life. Since the brain may behave differently while people are actively situated in ecologically-valid environments versus highly-controlled laboratory environments, it remains unclear how well the current laboratory-oriented BCI demonstrations can be translated into operational BCIs for users with naturalistic movements. Understanding inherent links between natural human behaviors and brain activities is the key to ensuring the applicability and stability of mobile BCIs. This study aims to assess the quality of steady-state visual-evoked potentials (SSVEPs), which is one of promising channels for functioning BCI systems, recorded using a mobile EEG system under challenging recording conditions, e.g., walking. To systematically explore the effects of walking locomotion on the SSVEPs, this study instructed subjects to stand or walk on a treadmill running at speeds of 1, 2, and 3 mile (s) per hour (MPH) while concurrently perceiving visual flickers (11 and 12 Hz). Empirical results of this study showed that the SSVEP amplitude tended to deteriorate when subjects switched from standing to walking. Such SSVEP suppression could be attributed to the walking locomotion, leading to distinctly deteriorated SSVEP detectability from standing (84.87 ± 13.55%) to walking (1 MPH: 83.03 ± 13.24%, 2 MPH: 79.47 ± 13.53%, and 3 MPH: 75.26 ± 17.89%). These findings not only demonstrated the applicability and limitations of SSVEPs recorded from freely behaving humans in realistic environments, but also provide useful methods and techniques for boosting the translation of the BCI technology from laboratory demonstrations to practical applications.

## Introduction

Recent advances in mobile electroencephalogram (EEG) technologies (Stopczynski et al., 2011; Wang et al., 2011; Chi et al., 2012) have radically boosted the demand of building mobile EEG-based brain-computer interfaces (BCIs) for various real-life applications, such as entertainment and clinical/in-home monitoring, assessment and rehabilitation. Thus, understanding and characterizing inherent links between human behaviors and EEG dynamics are the cores of dominating the applicability of mobile BCIs. Over the past decades, considerable laboratory-oriented BCI studies/demonstrations have led to fundamental and practical insights into how human brain actively/passively reacts in a closed-loop BCI. However, both theoretical and exploratory evidences suggest that brain dynamics might behave distinctively in response to natural environments versus those observed in highly-controlled laboratory environments (Mcdowell et al., 2013). For instance, the brain switches to a different operating method while humans actively behave, move, walk, and orient in ecologically-valid environments (Gramann et al., 2011). Sparse studies have devoted to explore the performance of applying a closed-loop BCI in real-world environment (Kohlmorgen et al., 2007; Blankertz et al., 2010). It remains unclear how well the current laboratory-oriented demonstrations can be translated into operational BCIs for users under their natural head/body positions, postures and movements. This translation can facilitate the use of operational BCI systems at patient’s home (Sellers et al., 2010). Therefore, unveiling the brain dynamics associated with naturalistic human behaviors is of great interest and urgent in effective translational neuroscience.

A steady-state visual evoked potential (SSVEP)-based BCI falls into the category of reactive BCI that derives its outputs from the brain activity in reaction to external stimulation (Zander and Kothe, 2011). For a clear comparison, among active, passive and reactive BCIs, please see Zander and Kothe, 2011. SSVEP, along with evoked potentials, event-related potential (ERP), and sensorimotor rhythms (Wolpaw et al., 2002), is widely adopted in current active and reactive BCIs. The SSVEP signal is a frequency-coded brain response that is generated as neurons of visual cortex synchronizing their firing to the frequency of continuous, repetitive visual stimulation. As the natural characteristics of SSVEPs, electrodes placed at the occipital region over the visual cortex can measure SSVEPs with high signal-to-noise ratio (SNR; Lin et al., 2006; Wang et al., 2006; Friman et al., 2007). Herrmann (2001) reported that SSVEP amplitudes are sensitive to the frequencies of visual flickers with predominant resonance peak at 30–80 Hz. Wang et al. (2006) further explored three subsystems that existed in SSVEP resonances with a major peak around 15 Hz, followed by two other peaks at 31 Hz and 41 Hz. On the other hand, by means of assigning a unique flickering frequency to each of visual targets, several laboratory studies (Calhoun and Mcmillan, 1996; Cheng et al., 2002; Kelly et al., 2005a; Wang et al., 2006; Muller-Putz and Pfurtscheller, 2008) have successfully demonstrated that SSVEP signals can serve as a communication carrier in actuating BCI systems with advantages such as high SNR, brief user training, and less individual difference. However, the previous studies all assessed SSVEPs from stationary and tethered individuals, who were instructed to avoid gross task-irrelevant head/body movements. One can expect that when mobile BCIs are deployed to freely moving and non-tethered users in the real world, the SSVEP-based BCI systems fully based on laboratory evidences of SSVEP characteristics might suffer from the generalizability issue. To date, little is known about the dynamics of SSVEPs accompanying naturalistic movements.

The unavailability of ease-of-use EEG sensing systems that do not require application of conductive gels to the scalp has long hindered BCIs from effective real-life applications. Novel mobile EEG systems, featuring wireless telemetry and/or non-prep dry electrodes, may significantly facilitate EEG recordings during natural movements and behaviors. Several studies have proved the efficacy of using either experiment-grade (Popescu et al., 2007; Wang et al., 2010, 2011; Zander et al., 2011; Chi et al., 2012) or consumer-grade (Campbell et al., 2010; Crowley et al., 2010; Bobrov et al., 2011; Petersen et al., 2011) mobile EEG systems in fundamental researches and BCI demonstrations. Furthermore, the lightweight head-mounted display devices have gained increasing attentions nowadays and enable an easy access to multimedia content at anytime and anyplace. Once the mobile EEG system is integrated with the display device in near future, a ubiquitous BCI system functioning in our real life becomes feasible. Nevertheless, until recently only scattered studies (Debener et al., 2012; Lin et al., 2013) employed such mobile EEG sensing technology to field recording. Thus, fully testing the capability and limitations of the mobile EEG/BCI technology is necessary not only for the practical generalizability issue, but also for any demands that involve brain activity monitoring of unconstrained, freely-moving subjects performing ordinary tasks in their living environments.

This study aimed to address the feasibility of using a mobile and wireless EEG system to decode SSVEPs during steady walking. To systematically explore the effects of walking locomotion on the SSVEPs, this study instructed participants to stand or walk on a treadmill running at speeds of 1, 2, and 3 mile(s) per hour (MPH) for eliciting different degrees of head/body movements while subjects were performing visual tasks. The main focuses of offline data analyses are: (1) evaluating the SSVEP quality using a mobile EEG system; (2) assessing the impact of walking locomotion on SSVEP signals; and (3) optimizing the SSVEP detection pipeline for moving humans. This study devoted to facilitate the real-life SSVEP-based BCI applications for freely behaving humans using a mobile EEG system.

## Materials and methods

### Participants

Nineteen healthy participants (14 males and 5 females; 24–33 years of age; mean age, 27.11 years) with normal or corrected-to-normal vision participated in this study. UCSD Human Research Protections Program approved this study. Each participant read and signed an informed consent before the experiment.

### Experiment setup

**Figure 1:**
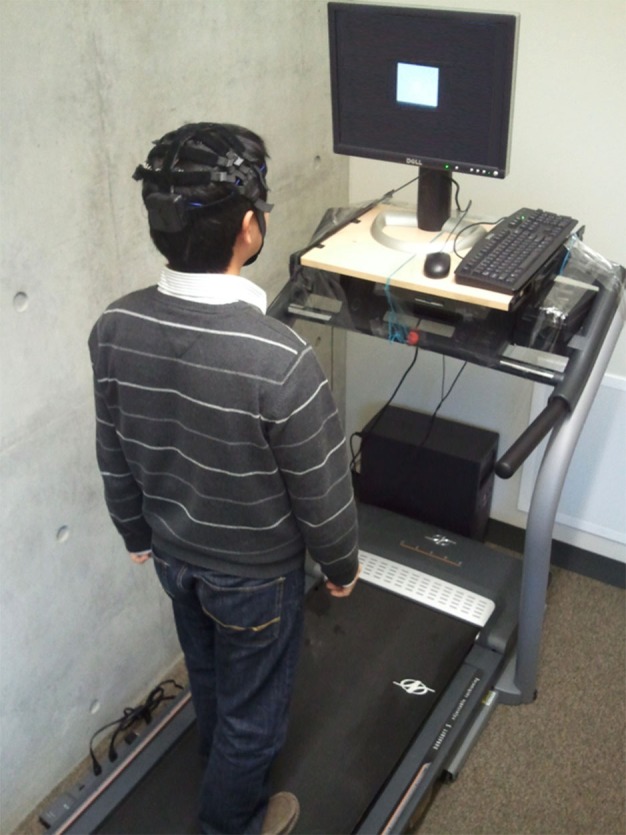
**The illustration of experiment setup for SSVEP recordings**.

To evaluate the impacts of walking locomotion on EEG/SSVEP signals, this study instructed participants to walk on a treadmill with three speeds of 1, 2, and 3 MPH. Participants were asked to attentively gaze at continuous, repetitive black/white visual flickers at the frequency of 11 or 12 Hz for 60 s while walking (Figure 1). The frequencies of the stimuli were in the high-frequency α-band because SSVEPs in this frequency range often lead to higher classification performance than other frequency bands (Gao et al., 2003). The higher SNR in the high-frequency α-band can be explained by higher SSVEP amplitudes and a concurrent suppression of spontaneous α-activities (Birca et al., 2006). In addition, the conditions of standing still on the treadmill and/or gazing at the screen with a black background were included for comparison. This study adopted the frequency approximation approach (Wang et al., 2010) to present single flicker (7.5 cm × 6.0 cm) on the center of a 19″ LCD monitor with a refresh rate of 60 Hz. The monitor was placed above the treadmill control panel and adjusted so that the flicker located in the center of each participant’s visual field. The participants were instructed to hold the treadmill hand grip during standing and walking, facing the monitor at a distance of 60 cm away. Each participant underwent the experiment consisting of 12 sessions (four treadmill speeds × three visual targets without counterbalancing) with a between-session rest of 10–20 s to prevent visual and/or motor fatigue.

### EEG data acquisition

This study adopted a 32-channel EEG system (Cognionics, Inc.) featuring soft fabric dry electrodes (Chi et al., 2013) and wireless

telemetry to sample EEG signals with 250 Hz. Notably, only two four-electrode straps (eight electrodes: P3, P1, P2, P4, PO3, PO1, PO2 and PO4) over the parietal and occipital areas were used in data recording. For assessing the quality of EEG signals using dry electrodes, two disposable electrodes with wet gel placed at O1 and O2 were also included. Both dry and wet electrodes were referenced to the same electrode placed at the forehead. Thus, this study used a total of 10 electrodes in the EEG recordings.

### Offline EEG data analysis

To assess the quality of SSVEPs for moving humans using a mobile EEG headset, an offline analysis was conducted to address three issues: (1) evaluating reliability and quality of SSVEP recorded by the non-prep and mobile EEG system; (2) exploring the impact of walking locomotion on the SSVEP signal quality; and (3) optimizing the SSVEP detection pipeline during walking movements.

#### EEG power spectral density

For each of 19 participants, this study collected a dataset of 12 10-ch 60-s EEG segments (four treadmill speeds × three visual tasks). This study adopted a semi-automatic artifact removal procedure to remove EEG artifacts induced by motion. The EEG data were first filtered by a 1–50 Hz band-pass finite impulse response (FIR) filter with zero phase-shift to remove the DC-drifts and high-frequency artifacts. Then, transient artifacts and noisy channels accounting for walking locomotion were sequentially removed by hand. Since the number and locations of noisy dry channels might vary from one subject to another. It was difficult to find an identical pair of dry channels for all subjects to make a wet-dry comparison. Alternatively, two dry electrodes closest to the wet electrodes (O1 and O2) were selected for each subject from the four parieto-occipital electrodes (PO1, PO2, PO3, and PO4). The data from seven participants were discarded for the spectrum analysis by subjective inspection. Two participants had poor signal quality at both of the two wet electrodes, and five participants had poor signal quality at the parieto-occipital electrodes. This study then applied the short-time Fourier transform (STFT) with a 250-point and 50% overlapping Hamming window to each of 60-s EEG segments to estimate the EEG spectrogram with a frequency resolution of 1 Hz. The averaged power spectral density (PSD) of each channel was derived by averaging the PSDs from different time windows. Lastly, this study employed a relative PSD, i.e., the ratio of PSD and the sum of total power (1–50 Hz), to compare the spectral characteristics in different conditions.

#### Offline steady-state visual-evoked potential (SSVEP) analysis

Previous SSVEP studies conducted with stationary, movement-constrained subjects have demonstrated several factors that affected the performance of SSVEP detection, including detection algorithm, data length, and channel montage (Lin et al., 2006; Wang et al., 2006; Bin et al., 2009). The offline SSVEP analysis of this study aimed to explore the effects of these factors on the much challenging datasets and explore an optimal data-processing pipeline for detecting 11 and 12 Hz SSVEPs collected from freely moving subjects in a naturalistic environment. First, this study implemented and compared PSD-based analysis (PSDA) and canonical correlation analysis (CCA; Lin et al., 2006) algorithms commonly used in SSVEP-based BCIs. Second, to evaluate the optimal data length, each of eight 10-ch 60-s visual-induced EEG trials (four speeds × two flickering frequencies) was then segmented into non-overlapping *N*-s epochs (*N* = 1–5) for comparison. Third, this study tried to explore the optimal channel montage from eight dry electrodes for each detection method.

PSDA is the most widely used frequency-detection method in early BCI implementations. PSD is estimated within a given time window of EEG data. The PSDA method decides the frequency of an SSVEP signal according to the peak of spectral amplitude. This study used the PSD values at 11 and 12 Hz as features for target identification. The frequency with higher PSD value was

considered as the target frequency. Using prolonged EEG data for deriving the spectra can increase the SNR (Wang et al., 2006) and thereby improve the SSVEP detectability (Lin et al., 2006; Bin et al., 2009). Since the PSDA method can be conducted on a single channel or bipolar channels, the advantages of low computational cost and less electrode requirement lead to an irreplaceable role in BCI applications. The STFT with a non-overlapping 250-point Hamming window was applied to *N*-s EEG epochs to estimate the PSD over time with frequency resolution of 1 Hz. This study adopted a bipolar-channel montage for the PSDA calculation towards better SNR. In an optimal bipolar measurement of SSVEPs, most of the spontaneous background activities in the two electrodes are eliminated while the SSVEP component is retained (Wang et al., 2006). Notably, since the optimal channel montage may vary by subject, this study performed an exhaustive search for optimal bipolar channels, based on the criterion of maximal frequency detection performance, from the eight dry electrodes for each subject.

Unlike the frequency-based PSDA method, CCA is a multivariate statistical method that aims to maximize the correlation between the linear combination of multichannel EEG signals and the combination of sinusoidal templates (sine and cosine waves for automatic phase adjusting) corresponding to the targeted flickering frequencies (Lin et al., 2006; Bin et al., 2009). The SSVEP frequency is determined according to the maximal canonical correlation among the predefined template frequencies. For example, the CCA method returns the SSVEP frequency of 11 Hz if the correlation coefficient between the measured signals and the 11 Hz template is larger than that between the measured signals and the 12 Hz template. The CCA calculation that uses channel covariance information has been suggested to return SNR-enhanced SSVEP signals. Unlike PSDA, CCA does not require channel selection and its multivariate statistical analysis makes it capable of improving the SNR of SSVEPs through spatial filtering. Note that CCA calculation in this study only relied on the fundamental frequency of template signals, because previous study has shown the inclusion of harmonics did not significantly improve the SSVEP detection (Bin et al., 2009). In addition, the CCA calculation was conducted on several montages from eight dry channels for comparison, including using all channels (eight-Ch), four parietal channels (P-4Ch: P3, P1, P2 and P4), four parieto-occipital channels (PO-4Ch; PO3, PO1, PO2 and PO4), two lateral parieto-occipital channels (LPO-2Ch; PO3 and PO4), and two inferior parieto-occipital channels (IPO-2Ch; PO1 and PO2). Note that the channel montage IPO-2Ch that is closed to the wet electrodes (O1 and O2) was used to perform the wet-dry electrode comparison.

To perform the CCA-PSDA comparison in a realistic online fashion (Lemm et al., 2011), this study selected an optimal bipolar channel for PSDA by estimating detection accuracy with a two-fold cross validation. The training trials were only used to perform the exhaustive channel search for PSDA, whereas the test trials were adopted to calculate frequency detection performance.

In sum, this study systematically performed both PSDA and CCA methods on *N*-s EEG epochs with different channel montages. The SSVEP frequency was calculated according to the maximal PSD value (in PSDA) and correlation coefficient (in CCA) between 11 Hz and 12 Hz. This study aimed to explore an optimal pipeline for improving SSVEP detectability in moving humans. The detectability is the percentage of correctly detected epochs in frequency detection and was only calculated in the sessions in the presence of visual flickers (11 Hz and 12 Hz). The conditions without visual stimuli were only used for evaluating EEG spectral fluctuations irrelevant to visual stimulation.

## Results

### EEG spectral fluctuations associated with different walking speeds

**Figure 2:**
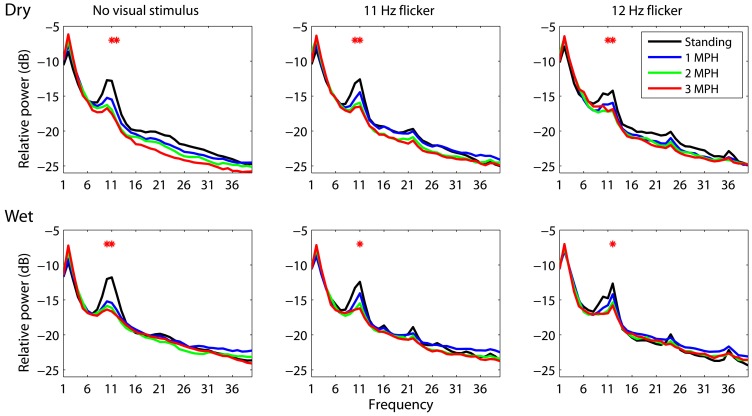
**The dry-wet electrode comparison in spectral fluctuations associated with different walking speeds while presenting without/with visual flickers (11 or 12 Hz)**. The asterisk indicates the significant difference between speeds (*p* < 0.05).

An attempt of this study is to assess whether or not a mobile EEG system featuring dry electrodes is capable of acquiring laboratory-quality EEG signals in moving humans. To this end, this study performed the wet-dry electrode comparison using spectral characteristics associated with standing and walking locomotion. This study employed the analysis of variance (ANOVA) to reveal the impact of different walking speeds (standing, 1 MPH, 2 MPH, and 3 MPH) on spectral changes along frequency (1–50 Hz). Figure 2 depicts EEG spectral fluctuations associated with different walking speeds using dry and wet electrodes. As subjects started walking, both types of electrodes presented comparable tendencies in α (8–13 Hz) suppression compared to standing still (black solid line). Walking speed more and less positively correlated with the degree of α-suppression. There was a statistically significant α-suppression (*p* < 0.05) at 11 and 12 Hz for both electrodes. The walking-related α-suppression was reproduced when subjects gazed at visual flickers during walking. In standing condition, both dry and wet electrodes detected resonance peaks at the stimulus frequencies (11 and 12 Hz) and the second harmonics (22 and 24 Hz). The third harmonic was only evident in the 12 Hz condition. The SSVEP amplitudes at the fundamental frequencies measured by both types of electrodes dropped significantly during walking (*p* = 0.05). Regardless of the presence or absence of visual flickers, either dry or wet electrodes exhibited a monotonic power increase at 2 Hz as walking speed increased.

**Figure 3:**
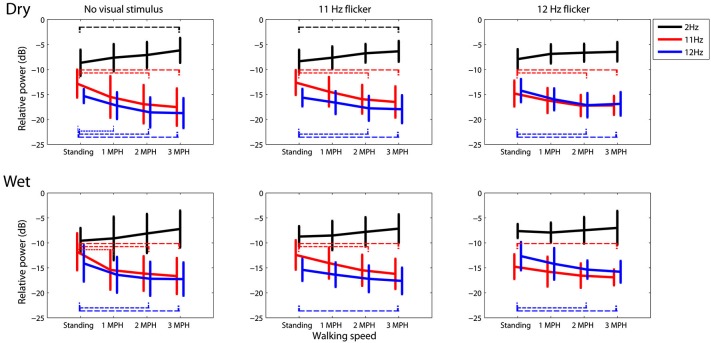
**The dry-wet electrode comparison in spectral fluctuations at 2, 11, and 12 Hz while subjects standing or walking on the treadmill with or without the presence of visual flickers (11 or 12 Hz)**. The thin dash line indicates the significant difference between speeds (*p* < 0.05), whereas the error bars represent the standard deviation of the results.

Figure 3 portrays the trend of the spectral changes at 2, 11 and 12 Hz at different walking speeds. In general, for either dry or wet electrodes faster walking locomotion accompanied a progressive spectral increase at 2 Hz, but a monotonic decrease at 11 and 12 Hz, regardless of the presence or absence of visual tasks. A *t*-test was performed to compare the mean spectral power between walking speeds. The results showed that in most of the cases the walking speed increased by two or plus miles per hour, e.g., from standing to 2 MPH or to 3 MPH, would lead to a statistically significant spectral differences (*p* < 0.05). Only dry electrodes measured a significant 2 Hz spectral augmentation at 3 MPH versus standing.

### Offline SSVEP analysis

The offline SSVEP analysis aimed to not only evaluate the feasibility of using a mobile EEG system to acquire SSVEP signals, but also explore the optimal parameters for SSVEP detection in moving humans. Several analyses were performed with an emphasis on: (1) SSVEP detectability in dry versus wet electrodes; (2) optimal electrode montage; (3) SSVEP detection algorithm; and (4) frequency sensitivity in SSVEP detection (11 vs. 12 Hz).

**Figure 4:**
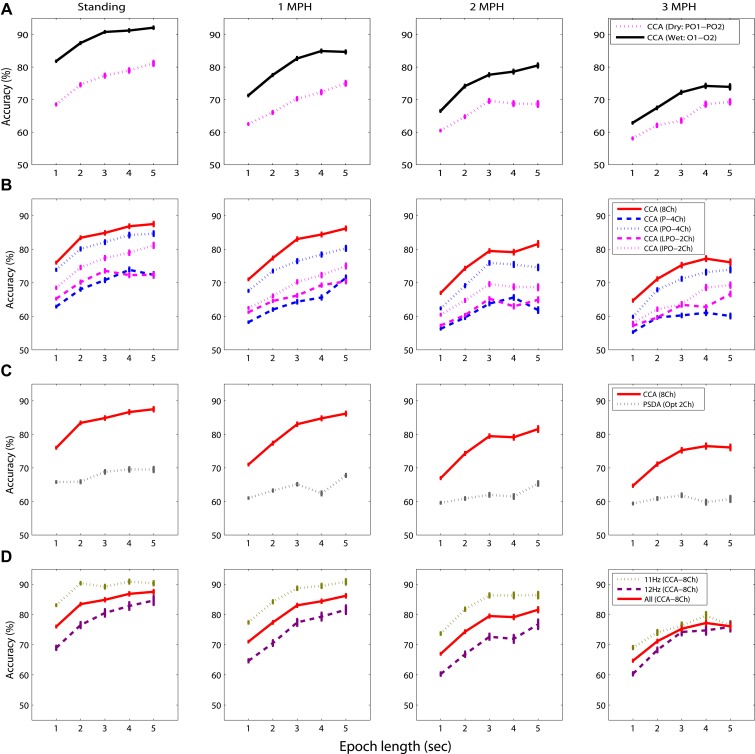
**The comparative results for evaluating the factors dominating the SSVEP detectability while standing still or walking with different speeds (1–3 MPH), including **(A)** electrode type, **(B)** channel montage, **(C)** detection algorithm, and **(D)** sensitivity of SSVEP frequency using different epoch lengths (1–5 s).** The error bars represent the standard error of the results.

Figure 4 shows the SSVEP detectability using different epoch lengths at different walking speeds. In general, SSVEP detectability was improved with prolonged EEG epoch under different walking speeds, and the detectability declined as walking speed increased. Specifically, Figure 4A shows the wet-dry comparison of CCA-based SSVEP detectability, i.e., wet electrodes (O1 and O2) vs. adjacent dry electrodes (PO1 and PO2). The results indicated that the detectability using wet electrodes (solid line) outperformed that using dry electrodes (dotted line) by at least 10% with different epoch lengths for the standing condition. SSVEP detectability decayed as walking speed increased from 1 to 3 MPH for both electrode types. The detectability decay was more evident in wet electrodes, leading to around 5% decrease per MPH increase, making wet and dry electrodes competitive at higher walking speeds. Figure 4B systematically assesses the CCA-based SSVEP detectability using different montages of dry electrodes. The result showed that using more channels (from 2 to 8) in general improved SSVEP detectability along different epoch lengths and under different walking speeds, except for the montage of using four parietal channels (P-4ch, blue dash line). The maximal accuracy was obtained by using 8 channels at any given walking speed, followed by using four parieto-occipital channels (PO-4ch, blue dotted line), two inferior channels (PO1 and PO2, pink dotted line) and lateral channels (PO3 and PO4, pink dashed line) of the parieto-occipital strap, and four parietal channels (P-4ch, blue dashed line). Interestingly, both 2-ch montages returned comparable or even better results than the montage of four parietal channels. The SSVEP detectability tended to decrease as walking speed increased no matter how many channels were involved in the analysis. Figure 4C illustrates the CCA-PSDA comparison in SSVEP detectability based on eight dry electrodes. The profiles along different epoch lengths showed that CCA apparently outperformed PSDA under all walking speeds. Lastly, Figure 4D shows the frequency sensitivity in SSVEP frequency detection (11 vs. 12 Hz) using the 8-ch CCA method under different walking speeds. The result indicated that the SSVEP detectability at 11 Hz (dotted line) was clearly higher than 12 Hz (dashed line) until the speed reached 3 MPH. The SSVEP detectability of 11 and 12 Hz was nearly identical during fast walking (at 3 MPH).

**Figure 5:**
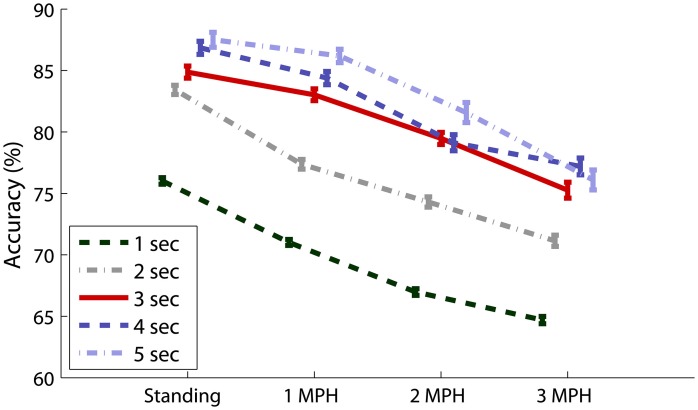
**Eight-ch CCA-based SSVEP detectability using different epoch lengths (1–5 s) under different walking speeds.** The error bars represent the standard error of the results.

Figure 5 overviews the impacts of different data lengths in 8-ch EEG epochs on the CCA-based SSVEP detectability. The result indicated that although the detectability improved using longer data epoch, there was no statistically significant difference (*p* > 0.05) after adopting epoch length longer than 3 s across all walking speeds. The use of 8-ch 3-s EEG epochs (solid line) in CCA obtained accuracy of 84.87 ± 13.55% for standing, which declined as subjects started walking (1 MPH: 83.03 ± 13.24%, 2 MPH: 79.47 ± 13.53%, and 3 MPH: 75.26 ± 17.89%).

## Discussion

Most of BCI demonstrations were conducted within well-controlled settings where tethered subjects had highly restricted movements. It remains unclear how well the laboratory-oriented demonstration can be translated into operational BCIs for users situated in real environments. This study aimed to assess the applicability of using a mobile EEG system to decode SSVEP signals in moving humans. The results showed that although the SSVEPs began to deteriorate while subjects engaged in faster walking locomotion, the obtained detectability from a conceptual BCI paradigm showed its potential in naturalistic environments outside highly controlled laboratory environments. Most importantly, this study found that the targeted brain responses that serve as BCI channels (e.g., SSVEPs in this study) would be more and less susceptible while human are actively behaving in real-life environments. This evidence confirmed that brain dynamics might behave distinctively in natural environments versus laboratory environments (Mcdowell et al., 2013).

Accordingly, prior to deploy a real-life mobile BCI, the desired BCI channel should be fully explored and characterized beyond the laboratory settings.

### Using a mobile EEG headset for moving humans

This study aimed to elucidate whether or not the non-prep, dry electrode and the mobile EEG headset provide acceptable quality of SSVEP signals in moving humans. To clarify this issue, this study used two wet electrodes placed at O1 and O2 for comparison. As shown in the wet-dry detectability comparison (c.f. Figure 4A), despite the accuracy using both electrode types deteriorated as the walking speed increased, the wet electrode tended to produce better accuracy for standing (by 10%) and different walking speeds (by 4%) using different epoch lengths. However, it is worth mentioning that due to the non-identical channel locations (wet: O1 and O2; dry: PO1 and PO2) used in the comparison, the detectability gap might not be fully attributed to the electrode types. It could be partially attributed to the fact that the occipital electrodes over the visual cortex have better SNR than those at the parieto-occipital areas (Wang et al., 2006; Lin et al., 2012). The 4-ch comparison (c.f. Figure 4B) also mirrored this phenomenon. That is, the parieto-occipital strap (PO-4ch) significantly outperformed the parietal strap (P-4ch) by 4–13% across different walking speeds and different epoch lengths. In addition, one might argue that the signal deviation of dry electrodes might be more vulnerable to movement interference (Guger et al., 2012). In our study, the dry electrodes tended to be significantly affected by 2 Hz artifacts during fast walking. The SSVEP fluctuations (11 and 12 Hz) measured by both electrodes under different walking speeds were comparable (c.f. Figure 3). Considering the practical factor such as ease-of-use for BCI users, as well as acceptable performance derived from multiple channels for moving humans (c.f. Figure 4B), using a mobile EEG system (dry, non-prep sensors) to record EEG/SSVEP signals under hostile recording settings should be feasible and practical for real-life BCI applications.

### Spectral dynamics associated with walking locomotion

The SSVEP signals (11 and 12 Hz) in this study were found to progressively decrease as the walking speed increased from 0 (standing still) to 3 MPH (c.f. Figures 2, 3). Two factors might contribute to the deterioration of SSVEPs in walking locomotion. First, the SSVEPs targeted within the α-range (8–13 Hz) might be highly constrained by the α-suppression attributed to the transition from idling to alert state. The people who are awake and engage no processing of sensory input and motor execution typically exhibit dominant 8–12 Hz resting EEG activity, called idling activity. One major idling activity, the α-rhythm over the visual cortex, can be inhibited by the increase of visual processing during walking (Williamson et al., 1997). The behavior of the idling activity may very likely explain in part the resulting occipital α-attenuation in this study. This study further explored that the level of deterioration was positively correlated with the intense and speed of walking locomotion, generally resulting in a significant drop while speeding the walking steps, especially for dry electrodes (c.f. Figure 3). Since the SSVEP signal is assumed to arise from stimulus-induced phase resetting of ongoing EEG oscillations (Sauseng et al., 2007), it is reasonable to assume that the suppression of spontaneous α-rhythm led to reduced SSVEP amplitudes during fast walking. Second, participants reported certain visual distraction while keeping up the movement of the treadmill, especially for the speed of 3 MPH. Since visual spatial attention plays an important role in modulating the SSVEP magnitude (Morgan et al., 1996; Kelly et al., 2005a; Lin et al., 2012), the suppression of SSVEP signals could be also in part attributed to the loss of visual focus from the flickering stimulus and/or rapid bounce of visual focus due to head nodding. However, the result of this study was limited to further differentiate these two factors in the SSVEP suppression for moving humans.

Another interesting finding related to walking locomotion was the spectral augmentation at 2 Hz. The 2 Hz power tended to

monotonically increase as subjects started walking on the treadmill (c.f. Figures 2, 3), especially for dry electrodes, which might be more sensitive to motion artifacts. The head movement accompanying natural walking might explain this phenomenon. Our very recent study (Lin et al., 2013 under review) had demonstrated that the head movement especially for walking at 3 MPH majorly engaged an intense 2 Hz head nodding (recorded by a vertical gyroscope sensor). This 2 Hz head-nodding movement swayed the EEG headset, encapsulating cables and circuitry, and therefore yielded low-frequency drifts in EEG signals. Fortunately, the 2 Hz headset-swaying due to head nodding accompanied by gait cadence (tested up to 3 MPH in this study) did not deteriorate the quality of SSVEPs (11 and 12 Hz). However, it might considerably contaminate ERP signals, which are widely used in ERP-based BCIs (Wolpaw et al., 2002).

### Optimal parameters for SSVEP detection in moving humans

Several factors including data length, channel montage, decoding method, and SSVEP resonant frequency were reported to affect the performance of SSVEP-based BCIs. However, the previous comparative studies were all conducted on stationary subjects within laboratory settings. This study compared the effects of these critical factors on SSVEP detection in a hostile recording condition (e.g., walking). The goal of this study was not only to test whether or not previous statements on SSVEP parameters remain valid, but also to explore an optimal procedure for detecting SSVEPs in moving humans.

First, as revealed in Figures 4, 5, using prolonged epoch length improved the SSVEP detectability consistently under different walking speeds, which was in line with the previous studies (Lin et al., 2006; Wang et al., 2006; Bin et al., 2009). This was attributed to the fact that applying longer EEG data to spectrum estimation can enhance the SNR of SSVEPs and thereby increase its detectability (Lin et al., 2006; Wang et al., 2006; Bin et al., 2009). Second, regarding the montage selection (c.f. Figure 4B), by comparing the detectability using different montages (P-4Ch vs. PO-4Ch, LPO-2Ch vs. IPO-2Ch), electrodes placed toward the central occipital cortex improved SSVEP detection. The above findings were reasonable as it is in accordance with the fact that the cortical sources of SSVEPs mainly localize in primary visual cortex (V1) and in the motion sensitive areas (V5), along with minor contributions from mid-occipital (V3A) and ventral occipital (V4/V8) areas (Di Russo et al., 2007). V1 is specialized for processing information about static and moving objects. Adopting IPO-2Ch montage directly probed the V1 activation and might provide more informative signals compared to other sites. In addition, more channels covering the entire visual cortex enhanced detecting the SSVEP signals (Friman et al., 2007). CCA is a multivariate statistical method that determines the SSVEP frequency by maximizing the correlation coefficient of multichannel EEG signals and targeted reference signals. Applying CCA to multichannel SSVEP signals thus can improve SNR of SSVEP and benefit the SSVEP detection (Lin et al., 2006; Bin et al., 2009). Previous CCA studies performed on data collected from stationary subjects (Lin et al., 2006; Bin et al., 2009) reported that the CCA method significantly outperformed the PSDA method, which supported our findings in the CCA-PSDA comparison. Last, as explored in Figure 4D, decoding 11 versus 12 Hz SSVEP predominantly contributed to the overall detectability until walking speed reaching 3 MPH. This result indicated that the SSVEP detectability of moving humans was vulnerable to the resonant frequencies of visual flickers, which was consistent to the findings in stationary subjects (Herrmann, 2001; Kelly et al., 2005b; Wang et al., 2006; Lin et al., 2012).

To conclude, the SSVEP findings under the standing condition, i.e., movement-constrained, were comparable with the previous studies with stationary (and seated) subjects. This study further explored the SSVEP dynamics in subjects walking steadily on the treadmill from 1 to 3 MPH. The SSVEP detectability tended to progressively deteriorate as walking speed increased no matter what channel montage, detection method, and flickering frequency was used. Although longer EEG epoch did improve the detectability, it could reduce the practicality of an on-line BCI system by decreasing information transfer rate (ITR), an index for evaluating BCI performance, which correlates positively to detection accuracy but negatively to decision time (Wolpaw et al., 2002). In addition, this study found that an epoch length exceeding 3 s did not significantly improve the detectability for moving subjects. Accordingly, taking account of the montage generalizability, using 3-s 8-ch EEG data to the CCA decoder might be an optimal procedure to detect SSVEP signals in moving humans. Such protocol yielded acceptable accuracies of 75% ~ 83% in distinguishing binary SSVEPs (11 Hz and 12 Hz) for walking speeds below 3 MPH, compared to standing (84.87 ± 13.55%). The empirical findings of this study not only explored inherit characteristics and limitations of SSVEP of freely moving participants under realistic environments, but also boosted the development of conceptual BCI paradigms that can be further translated to practically feasible systems.

### Implementation of an online BCI

The offline classification used in this study demonstrated the feasibility of a conceptual SSVEP BCI during walking. To implement an online BCI, the following major issues need to be addressed: (1) multiple stimuli with different flickering frequencies need to be presented simultaneously on the screen; (2) the data processing procedures such as band-pass filtering must be causal and fast to satisfy real-time implementation; (3) automatic selection of parameters such as electrodes and data length; and (4) visual or auditory feedbacks need to be provided to the subjects in near real time. These issues can be resolved using the existing methodologies developed in current SSVEP BCIs (Wang et al., 2006, 2011; Bin et al., 2009).

The “loss of focus” is a major challenge in building an online SSVEP-based BCI during walking. As discussed above, the deterioration of SSVEP amplitude during walking could be in part attributed to the loss of focus. A further challenge in an online BCI is to eliminate the interference among multiple targets caused by loss of focus. On one hand, increasing the distance between neighboring stimuli can reduce the interference between stimuli in the central and peripheral visual fields. On the other hand, a wearable stimulator (e.g., head-mounted display) may be used to facilitate fixation during walking.

### Future directions

Future efforts in decoding SSVEPs for freely moving humans can be devoted to elicit SSVEPs outside the α-frequency band, which is subject to the changes of visual processing during walking. Several studies have reported that the SSVEP resonance appeared at higher frequency band up to γ-band (30–50 Hz) (Herrmann, 2001; Wang et al., 2006; Lin et al., 2012). In addition, one future work is to replicate the treadmill experiment in which the visual stimuli will be presented through a head-mounted display device. This might help to elucidate the α-suppression attributed to the loss of visual attention and the engagement of walking locomotion. More importantly, the integration of a mobile EEG headset and a head-mounted display device might help to establish ubiquitous mobile BCI systems in ecologically valid environments. Similar to the SSVEP signals, the visual focus also strongly influences ERP amplitudes and in turn affects the performance of a gaze-dependent BCI speller (Treder and Blankertz, 2010). Another direction is to incorporate the gaze-independent paradigms (Treder et al., 2011; Riccio et al., 2012), which are applicable to patients with oculomotor impairments, to solve this issue using the same mobile settings.

## Author contributions

Conceived and designed the experiments: Yijun Wang. Performed the experiments: Chun-Shu Wei, Yuan-Pin Lin. Analyzed the data: Yuan-Pin Lin, Yijun Wang, Tzyy-Ping Jung. Wrote the paper: Yuan-Pin Lin, Yijun Wang, Tzyy-Ping Jung.

## Conflict of interest statement

The research was conducted in the absence of any commercial or financial relationships that could be construed as a potential conflict of interest.
